# Microbiota-accessible carbohydrates enhance gut microbiota stability and antibiotic resilience through production of quorum sensing molecule AI-2

**DOI:** 10.1080/29933935.2026.2646055

**Published:** 2026-03-23

**Authors:** Robert Keskey, Rebecca Meltzer, Tiffany Toni, Sanjiv Hyoju, Ellen Cohn, Jessica Cao, Andrew Benjamin, Adam Lam, Alexander Zaborin, Olga Zaborina, John Alverdy

**Affiliations:** a Hiram C. Polk, Jr., MD, Department of Surgery, University of Louisville School of Medicine, Louisville, KY, USA; b Section of General Surgery, Department of Surgery, University of Chicago, Chicago, IL, USA; c Pritzker School of Medicine, University of Chicago, Chicago, IL, USA; d Department of Surgery, University of Michigan, Ann Arbor, MI, USA; e Department of Surgery, Endeavor Health, Evanston, IL, USA

**Keywords:** Gut microbiome, antibiotic exposure, dietary fiber, quorum sensing

## Abstract

Dietary fiber and fat shape the gut microbiota and human health, yet their role in modulating the response of the microbiota to antibiotics remains underexplored. We hypothesized that dietary fiber, independent of fat content, mitigates antibiotic-induced weight loss and diarrhea in a microbiota-dependent manner. Mice were fed refined diets varying in fat and fiber contents for 6 weeks, compared to a standard plant-based chow diet. Following antibiotic administration, fiber consumption independent of fat reduced diarrhea and weight loss. High-fiber diets increased Bacteroidetes and decreased Firmicutes and Proteobacteria prior to antibiotic exposure, all of which correlated with elevated cecal short-chain fatty acids (SCFAs). Fermentable fiber increased AI-2 quorum-sensing pathway activity and improved Firmicutes resiliency to antibiotics. Supplementation with AI-2 reduced antibiotic-induced weight loss in mice fed high-fat, low-fiber diets. These findings suggest that fermentable fiber alters the gut microbiota composition and function, enhancing microbial resiliency and host tolerance to antibiotics. Dietary supplementation with microbiota-accessible fiber increased AI-2 production, stabilized Firmicutes populations, and attenuated antibiotic-associated weight loss, independent of dietary fat content.

## Introduction

Recent exposure to antibiotics has been shown to independently increase the risk for the development of postoperative complications, including subsequent sepsis and mortality.^1^^,^^2^ In fact, a single dose of antibiotics has recently been demonstrated to have a durable effect on the presence of antibiotic-resistant organisms in the gut microbiota.^3^ Despite national campaigns that promote antibiotic stewardship, antibiotic prescribing continues to increase at an alarming rate. It is hypothesized that the risks associated with antibiotic exposure may be related to antibiotic-induced alterations within the intestinal microbiota.^4-6^ Antibiotic-induced changes in the microbiota can lead to compositional and functional changes in the microbiota, which correlate with impaired host health and increased infectious complications.^7^^,^^8^

Recently, diet has been shown to play a major role in the response and recovery of the intestinal microbiota after exposure to antibiotics.^7^^,^^9^^,^^10^ However, there is limited evidence that determines how the preantibiotic state of the microbiota affects the response of the host to antibiotic therapy. An improved understanding of how different diet-induced changes in the intestinal microbiota impact the response of the host to antibiotics, may help the discovery of interventions to reduce the risks associated with antibiotic exposure. Currently, most patients in the United States consume a Western diet that is low in fiber and high in fat. A western diet shapes the composition and function of the intestinal microbiota, resulting in a loss of health-promoting bacteria and decreased production of health-relevant metabolites (i.e., SCFAs).^11-15^ These western diet-induced alterations can have a devastating impact on the host, as the combination of a western diet and antibiotic exposure has been shown to have an additive impact on the microbiota.^7^ For example, the combination of a high-fat diet and ciprofloxacin exposure resulted in a significantly different impact on the composition and function of the microbiota when compared to a control low-fat diet.^7^ Similarly, we have shown that in mice fed a western diet and subjected to antibiotics and surgical stress developed post-operative lethal, gut-derived sepsis.^16^ These findings suggest that the resiliency of the intestinal microbiota is greatly reduced when mice are subjected to a low-fiber/high-fat western diet, which may have a detrimental effect on host health.

Owing to the variability of fiber sources in high-fat, commercially available diets, it remains unclear the extent to which the resiliency of the microbiota to antibiotics is a result of the reduction in fat or increase in fiber. Previous experiments have used refined low- and high-fat diets in which fermentable fibers, carbohydrates that are accessible by the microbiota for fermentation, are absent. Therefore, conclusions on the effect of dietary fat on microbiota composition have been made in the background of diets depleted in fermentable fiber.^17^ Fermentable fiber can be referred to as microbiota accessible carbohydrates (MACs), which are essential for maintaining health-promoting bacteria.^18^ MACs are resistant to digestion by the host, and are therefore available for utilization by gut bacteria, and provide a metabolic substrate that results in significant alterations in the community structure and metabolic activity of the microbiota.^18^ Therefore, the absence of fiber or lack of microbiota accessible fiber in experimental diets may result in a reduction of associated short-chain fatty acids (SCFAs), which play an essential role in host health and have a major impact on conclusions made in dietary experiments.^17^ Recent studies have demonstrated that the absence of fermentable dietary fiber, independent of dietary fat, results in a reduction in health-promoting Bacteroidetes and SCFAs.^17^ Compositional and functional changes to the microbiota induced by fiber depletion may impact the host immune system and increase pathogen colonization, both of which would be exacerbated by antibiotic treatment.^19^^,^^20^ To further implicate the importance of dietary fiber, a recent study demonstrated that the recovery of the intestinal microbiota after antibiotic exposure is significantly impaired when dietary fiber is removed.^9^

Here, we hypothesized that the content of microbiota accessible fiber within a diet, independent of its fat content, can impact the host response to antibiotics in a microbiota-mediated manner. Therefore, we aimed to define the dual roles of microbiota-accessible fiber and fat on the ability of the host to resist the negative impacts of antibiotic treatment. To test this, we designed customized diets that varied in fat, microbiota-accessible fiber (resistant starch, defined as a high-fiber diet), and microbiota-noninaccessible fiber (cellulose and corn starch, defined as a low-fiber diet) for consumption in a murine model of antibiotic exposure.

## Methods

### Mouse husbandry

Male C57BL/6 mice (Charles River Laboratory) aged 6 weekswere housed in temperature-controlled 12 h light/dark cycle rooms at the University of Chicago Animal Facility. Customized diets were developed in Tekland. The diets used varied in fat, microbiota-accessible fiber (resistant starch, defined as a high-fiber diet, HFb), and microbiota-noninaccessible fiber (corn starch, defined as a low-fiber diet, LFb). High- and low-fiber diets contain equal amount of carbohydrates. The mice were randomly assigned to *ad libitum* feeding on either: standard rodent chow (SD, Envigo, *n* = 20), high-fat/low-fiber diet (HF–LFb, Teklad TD.08811, *n* = 40), high-fat/high-fiber diet (HF–HFb, Teklad TD.190230, *n* = 20), low-fat/low-fiber diet (Teklad TD.120724, *n* = 20), or low-fat/high-fiber diet (LF–HFb, Teklad TD.190229, *n* = 20) for 6 weeks and weighed weekly. After 6 weeks of ad libitum consumption of their respective diets, the mice were administered antibiotics twice per day for 5 d, consisting of intraperitoneal (i.p.) cefoxitin (30 mg/kg, Hikma Pharmaceuticals, Eatontown, NJ) and oral (p.o.) clindamycin (70 mg/kg, Clindrops; Henry Schein, Dublin, OH). Following antibiotic administration, the severity of diarrhea was scored from 0–2, where 0 = no diarrhea, 1 = slight perianal erythema and loose stool, and 2 = severe perianal erythema and liquid stool. All the mice were sacrificed via carbon dioxide inhalation. Intestinal length was measured by removing the entire gastrointestinal tract en bloc from the distal esophagus to the anus, and the length of the small intestine, cecum, and colon were measured. All the experiments were conducted in accordance with National Institute of Health (NIH) guidelines, and approval was obtained from the University of Chicago Institutional Animal Care and Use Committee (IACUC protocol 71744).

### 16S rRNA gene amplicon sequencing and sequence data analysis

Microbial DNA extraction from the cecal contents and stool was performed using a power faecal DNA isolation kit (Qiagen, Carlsbad, CA) with 5 mice per diet group for both pre- and post-antibiotic exposure. For library preparation, DNA was amplified using the barcoded 12-bp Golay primer set designed for the Earth Microbiome Project (EMP). PCR was performed according to the manufacturer's protocol using the EMP primers, mPNA, AccuStart II PCR ToughMix, and extracted DNA (Quntabio). After amplification, the PCR products were quantified by using a PicoGreen dsDNA quantitation assay (Invitrogen). The results of the quantification were used to normalize the amount of DNA from the PCR product used for sequencing and ensure that each amplicon was represented evenly during sequencing. Finally, an aliquot of the final pool was taken, and the DNA was purified by using an Agencourt AMPure XP PCR* purification system (Beckman-Coulter). The samples were then run on an Illumina MiSeq at Argonne National Laboratory (150 bp × 2). The raw sequences are available through the NCBI SRA (Reference number: PRJNA83864, metadata supplemental file 1).

Qiime2^21^ was utilized for 16S rRNA gene sequence analysis, and the demux emp-paired-end command was used to demultiplex and join the paired-end reads. Quality filtering was performed using Deblur. Taxonomy and amplicon sequence variants (ASVs) were assigned using a Greengenes classifier 2022.10. The sequences were further analyzed utilizing the Phyloseq^22^ package within R. Samples were rarefied to a depth of 10,000 reads per sample. For the alpha diversity, the Shannon index was used. The Kruskal‒Wallis test followed by Dunn pairwise comparison with the Benjamin‒Hochberg correction was performed comparing alpha diversity between the dietary groups to determine significance. The beta diversity was analyzed using principal component analysis (PCoA) plots that were generated based on a weighted UniFrac dissimilarity matrix. Compositional plots were created utilizing *microshades* in R.^23^ PERMANOVA was utilized for statistical analysis of beta diversity.

PICRUSt2 (version 2.4.1)^24^ analysis was performed to predict microbiota function. The picrust2_out_pipeline function was utilized using a nearest sequence taxon index cutoff of 2. The contributions of samples and individual taxa to enzymes, designated by enzyme commission numbers, and pathways were determined utilizing KEGG designations. Taxon relative functional abundance was determined by the relative abundance of a given taxa multiplied by the number of gene copies present within the given taxa. The taxon relative functional abundance was compared across all significant taxa. STAMP was utilized to determine functional differences between the cecal and stool microbiota of the different diets pre- and post-antibiotic exposure.^25^ DESeq2 was utilized to determine the differential abundance of individual species of bacteria based on 16S sequencing.^26^

### GC–mass spectrometry

SCFAs were extracted from flash-frozen mouse feces, utilizing approximately 50 mg of mouse feces, using diethyl ether (Fisher Scientific), derivatized using N-tert-butyldimethylsilyl methyltrifluoroacetamide with 1% tert-butyldimethylchlorosilane (Sigma), and run on an Agilent Single Quad GC–MS (5977A Single Quad and 7890B GC). 4-Methylvalonic acid was spiked into each sample as an internal control and used to measure extraction efficiency. Standard curves were generated utilizing butyric acid, propionic acid, and acetic acid. All values are normalized to the sample mass, and the amounts of SCFAs were calculated on the basis of generated standard curves for each individual SCFA.

### Cecal microbiota AI-2 detection assay

The key quorum-sensing molecule, AI-2, was measured as previously described, utilizing the AI-2 reporter strain *Vibrio campbelli* (ATCC strain BB170), and *Vibrio campbelli* (ATCC strain BB152), which constitutively produces AI-2, was used as a positive control.^27^^,^^28^ Briefly, the cecal contents were isolated from the mice on their respective diets and resuspended in 0.1 M MOPS (pH 7.0). The samples were centrifuged and then filtered through a 0.2 µm filter. The filtrates were then vacuum-dried and resuspended in water at a concentration of 50% weight/volume. The concentrated filtrates were then added to the AI-2 reporter strain, and luminescence was measured overnight. Relative AI-2 production was normalized to the nadir of the negative control. Utilizing *E. coli* strains that constitutively produced AI-2 (ARO071, sm^R^, ΔlacIZYA:frt, Δgalk:Plac:yfp:amp, ΔlsrK:frt) and a control strain (ARO093, sm^R^, ΔlacIZYA:frt, Δgalk:Plac:yfp:amp, ΔlsrK:frt, ΔluxS:frt), we tested the sufficiency of soluble AI-2 to prevent antibiotic-induced weight loss in HFLFb-fed mice.^28^ Bacteria were grown at 37 °C until the log phase, and the supernatants were then collected and filtered through a 0.2 µm filter. The supernatant was administered via enema to HFLFb diet-fed mice the day prior to and during antibiotic treatment.

## Results

### Dietary fat content influences weight gain independent of fiber content, and dietary fiber content impacts intestinal tract length independent of fat content

**Figure 1.**
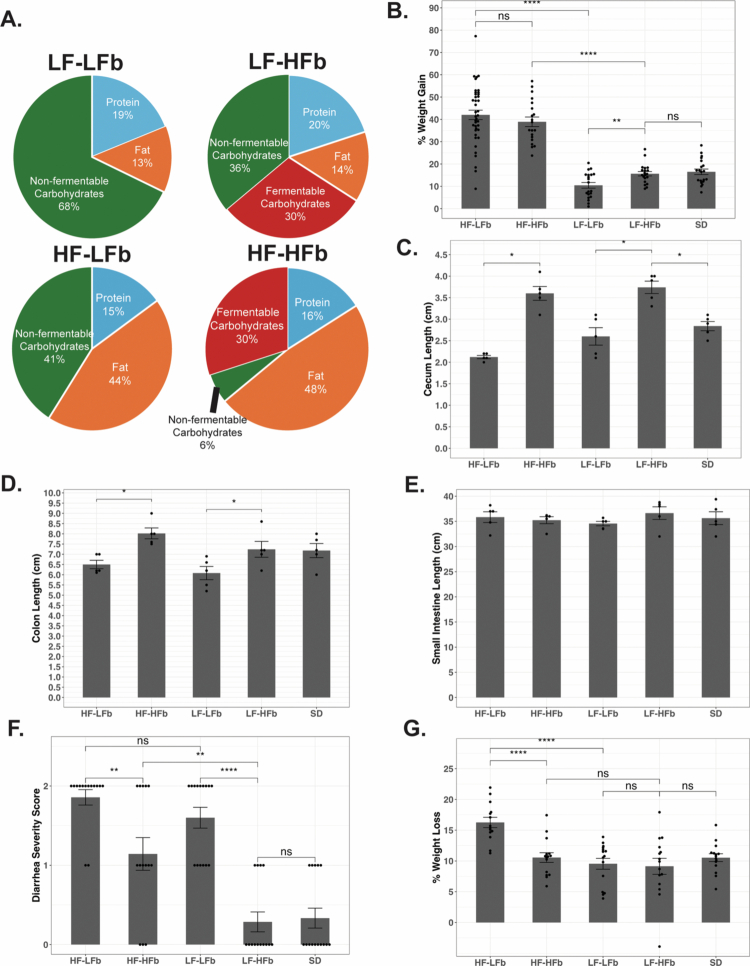
Fiber supplementation reduces antibiotic-induced diarrhea and weight loss. (A) Compositional make-up of the experimental diets where LF = low fat, HF = high fat, LFb = low fiber, and HFb = high fiber. (B) The percent weight gain after 6 weeks on each respective diet is demonstrated in the panel (HF–LFb *n* = 40, HF–HFb *n* = 20, LF–LFb *n* = 20, LF–HFb *n* = 20, SD *n* = 20). The impacts of the experimental diets on the cecum (C), colon (D), and small intestine (E) length were measured at the end of 6 weeks of feeding (*n* = 5 per diet). The mice were administered 5 d of antibiotics (p.o. clindamycin, i.p. cefoxitin), and diarrhea severity was scored from 0–2: 0 = no diarrhea, 1 = mild diarrhea, and 2 = severe diarrhea. Antibiotic-induced diarrhea (F), and weight loss (G) were compared between the respective diets after 5 d of antibiotics, *n* = 15 for all groups. *p* > 0.05 ns, *p < 0.05, **p < 0.01, ***p < 0.001, and ****p < 0.0001.

Mice were placed on their respective diets for a total of 6 weeks. Custom diets containing low-fat/low-fiber (LF–LFb), low-fat/high-fiber (LF–HFb), high-fat/high fiber (HF–HFb), and high-fat/low fiber (HF–LFb) were specifically designed and produced by Tekland at minimum mice per group pre- and post-antibiotic exposure (Figure 1A). As expected, the dietary fat content had a significant impact on weight gain at the end of 6 weeks when compared to SD group (Figure 1B). In the high-fat diets, fiber content did not result in significant changes in weight gain (42.0 vs. 38.9%, HF–LFb vs. HF–HFb). In the low-fat diet group, weight gain was similar to that in the SD group (Figure 1B).

As previously shown,^17^ fiber supplementation had a significant impact on the lower gastrointestinal tract, increasing the cecum length in both low-fat (2.6 vs. 3.7  cm, LF–LFb vs. LF–HFb, *p* ≤ 0.05) and high-fat (2.1 vs. 3.6  cm, HF–LFb vs. HF–HFb, *p* ≤ 0.05) diet-fed mice (Figure 1C). The addition of dietary fiber also resulted in a significant increase in colon length in both low-fat (6.1 vs. 7.2 cm, LF–LFb vs. LF–HFb, *p* ≤ 0.05) and high-fat (6.5 vs. 8.0 cm, HF–LFb vs. HF–HFb, *p* ≤ 0.05) diet-fed mice (Figure 1D) without impacting the length of the small intestine (Figure 1E).

### Dietary fiber attenuates antibiotic-induced diarrhea and weight loss

After 5 d of antibiotics, the severity of antibiotic-induced diarrhea was compared between diets and was rated on a 2-point scale as described above. The addition of microbiota-accessible fiber resulted in a significant attenuation of diarrhea severity in both high-fat (1.85 vs. 1.1, HF–LFb vs. HF–HFb, *p* ≤ 0.01) and low-fat (1.6 vs. 0.3, LF–LFb vs. LF–HFb, *p* ≤ 0.01) diet-fed mice (Figure 1F). No diarrhea developed in LF–HFb diet-fed mice, similar to SD-fed mice (Figure 1F). These results demonstrate that both high dietary microbiota-accessible fiber and low dietary fat can mitigate antibiotic-induced diarrhea. The combination of high-fat with low-fiber (HF–LFb) had the most drastic antibiotic-induced weight loss (Figure 1G), while there were no differences in all the other diets as compared with SD.

### Both dietary fat and fiber impact intestinal microbiota composition

**Figure 2.**
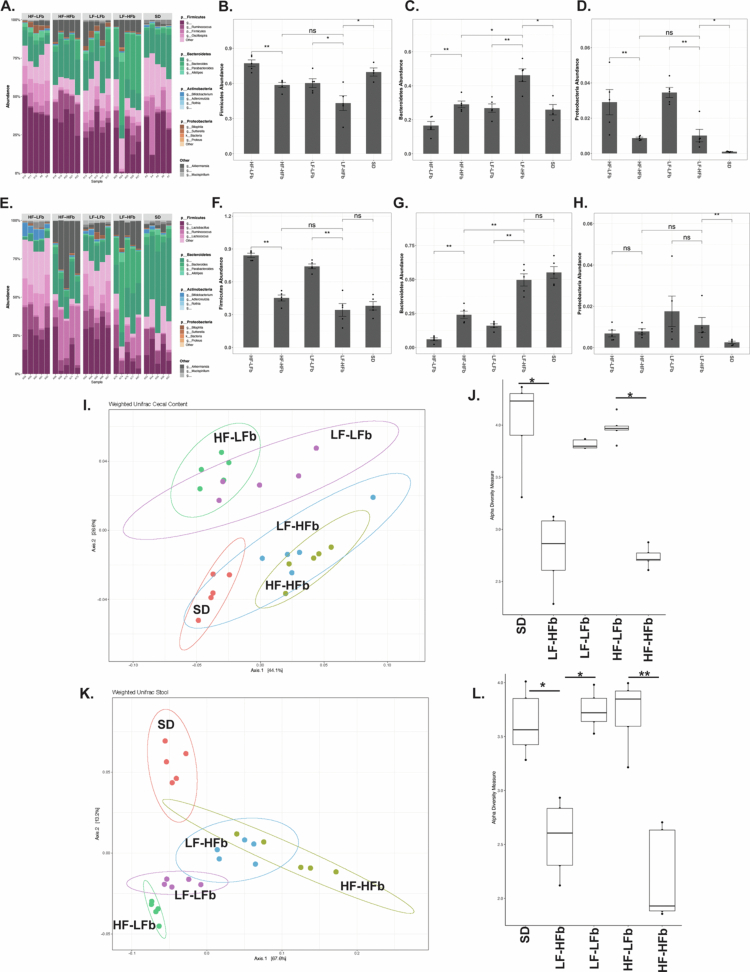
Dietary fiber significantly impacts the intestinal microbiota composition in both low- and high-fat diets. 16S rRNA sequencing was performed on both the cecal content and the stool prior to the administration of antibiotics. Dietary fiber significantly altered the composition of the cecum (A) and stool (E) in a similar manner. Both fat and fiber composition impacted the abundance of Firmicutes, Bacteroidetes, and Proteobacteria in both the cecum (B–D) and the stool (F–H). Beta diversity determined by weighted UniFrac resulted in clustering of both the cecum (I), and stool (K) microbiota in the presence of dietary fiber. The addition of dietary fiber resulted in a reduction in cecal (J), and stool (L) microbiota alpha diversity. *n* = 5 for all the groups. *p* > 0.05 ns, *p < 0.05, **p < 0.01, ***p < 0.001, and ****p < 0.0001.

The impact of the various diets on the intestinal microbiota was assessed using 16S rRNA gene amplicon sequencing analysis of expelled stool and cecal contents. Both the amount of dietary fiber and fat resulted in significant alterations to the microbiota composition within the stool and cecum (Figure 2A,E). Within the cecum (Figure 2B–D) and stool (Figure 2F–H), both the addition of fiber and the reduction of fat resulted in a significant increase in Bacteroidetes and a reduction in Firmicutes and Proteobacteria (*p* < 0.05). Similarly, the consumption of the low-fat diets (LF–LFb and LF–HFb) had significantly higher levels of Bacteroidetes (Figure 2C,G) and lower abundances of Firmicutes (Figure 2B,F) and Proteobacteria (Figure 2D,H) compared to the high-fat diets (HF–LFb and HF–HFb). When analyzed at the class level, there was a significant reduction in Gammaproteobacteria abundance in the stool when fiber was added to the diet (*p* = 0.02, Supplemental Figure 1). When beta diversity was assessed with weighted UniFrac, there was distinct clustering of the microbiota based on the dietary fiber present within the diet among both the cecal and stool microbiota (Figure 2I,K). The microbial compositions of the HF–HFb and LF–HFb diets clustered closer to those of the SD diet and were distinct from those of the HF–LFb and LF–LFb diets among both the cecal and stool microbiota (*p* = 0.001). Alpha diversity did not appear to be impacted by fat content but was reduced in the high-fiber diets (HF–HFb and LF–HFb) compared to the low-fiber diets (HF–LFb and LF–LFb) in both the cecum (Figure 2J) and stool (Figure 2L). The decrease in alpha diversity of the HFb diets may be explained by the single source of fiber present within the diets.

### Dietary fiber alters specific bacterial amplicon sequence variances (ASVs) and results in increased short-chain fatty acid production

**Figure 3.**
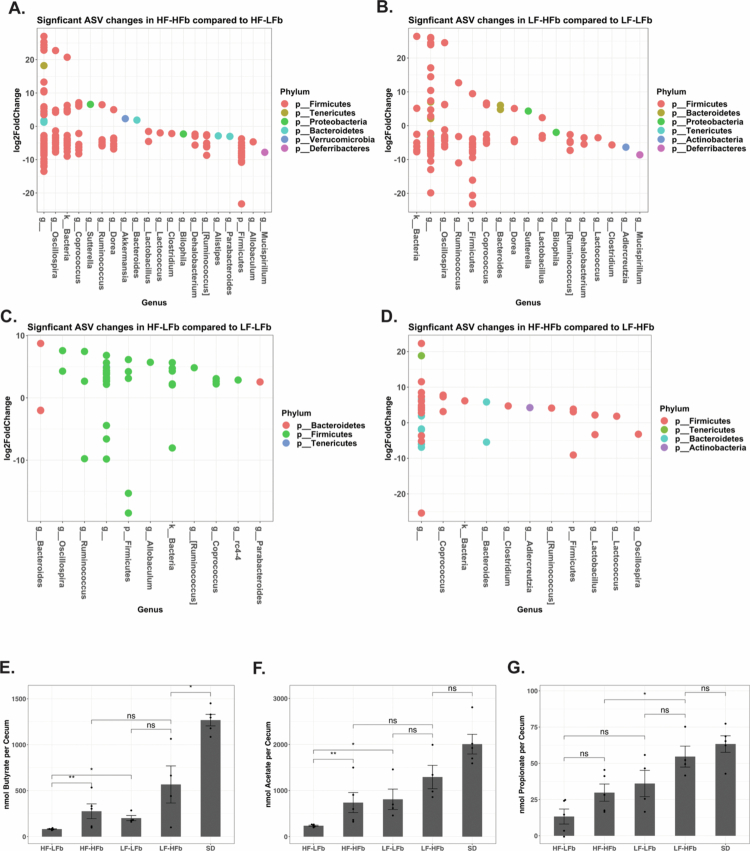
The addition of dietary fiber and fat significantly alters ASVs and cecal short chain fatty acids. On differential abundance analysis of 16S through DESeq2, high fiber content increased the abundance of Firmicute ASVs in high-fat (A) and low-fat (B) fed mice. Similarly, high fat content impacted the abundance of individual ASVs in both low-fiber (C) and high-fiber diets (D). GC‒MS was used to measure the levels of cecal SCFAs. The total amount of SCFA per cecum was compared between the respective diets. Cecal butyrate (E), acetate (F), and propionate (G) were measured. *n* = 5 for all the groups. *p* > 0.05 ns, *p < 0.05, **p < 0.01, ***p < 0.001, and ****p < 0.0001.

To determine the impact of the dietary interventions on intestinal microbiota function, differential abundance analysis was performed using DESeq2. The addition of fiber to both the high-fat (Figure 3A) and low-fat (Figure 3B) diets resulted in significant alterations in Firmicutes ASVs. Similarly, independent of fiber content, dietary fat also significantly impacted Firmicutes ASV abundance (Figure 3C,D). GC‒MS was utilized to measure SCFAs within the cecum prior to antibiotic treatment (Figure 3E‒G). SD-fed mice had significantly higher SCFAs production compared to the custom diets. In custom diets, the most significant difference in SCFAs was observed between mice that consumed a high-fat/low-fiber compared to a low-fat/high-fiber diet. The higher butyrate levels in the SD-fed mice compared to the custom high-fiber diet-fed mice may be a result of the custom high-fiber diets containing only a single source of microbiota-accessible carbohydrates compared to the multiple sources of microbiota-accessible carbohydrates present within standard rodent chow.

### Dietary fat and fiber significantly alter the cecal microbiota following antibiotic exposure

**Figure 4.**
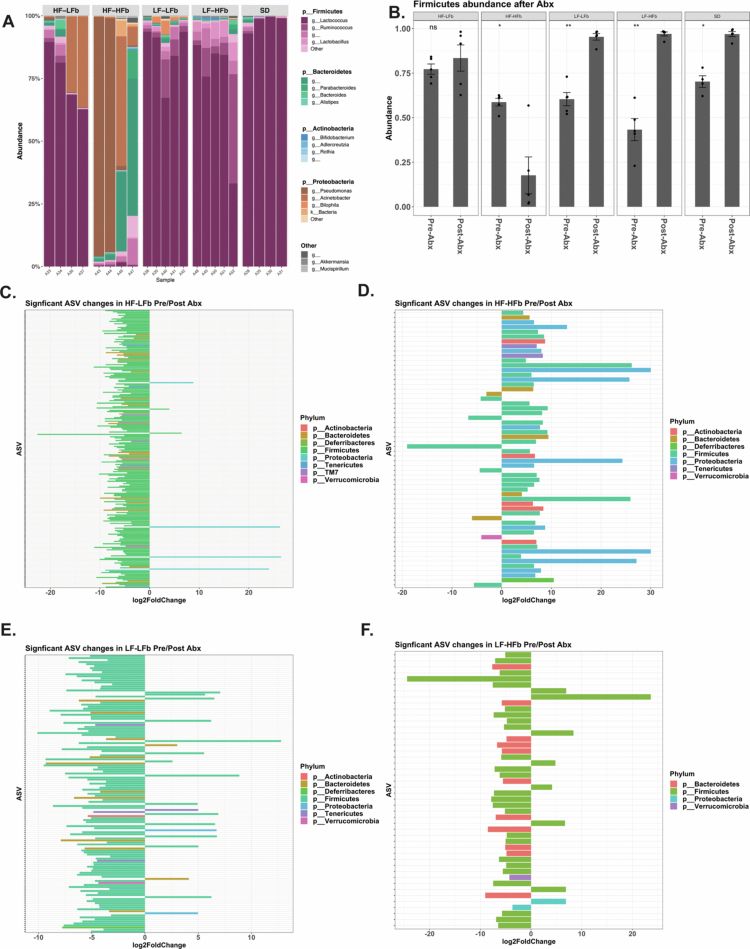
Dietary fiber maintains Firmicute abundance following antibiotic treatment. 16S rRNA sequencing was performed on the cecal microbiota following antibiotic treatment demonstrating differences in composition across diets (A) and a significant difference in Firmicute abundance dependent on dietary fat and fiber content (B). When preantibiotic and postantibiotic ASV abundances were compared, there was a significant reduction in ASVs from Firmicutes in both HF–LFb (C) and LF–LFb-fed mice (E), whereas HF–HFb (D), and LF–HFb (F) had fewer Firmicutes ASVs that were reduced by antibiotic treatment. *n* = 5 for all the groups. *p* > 0.05 ns, *p < 0.05, **p < 0.01, ***p < 0.001, and ****p < 0.0001.

To determine how antibiotics impact the cecal microbiota following antibiotic treatment, 16S rRNA sequencing was performed. When the cecal microbiota composition was compared at the phylum level, fat content appeared to impact the abundance of Firmicutes following antibiotic exposure (Figure 4A,B), with low-fat diets demonstrating a significant increase in overall Firmicutes abundance (Figure 4B). Differential abundance analysis was performed for specific ASVs comparing the cecal microbiota before and after antibiotic exposure for HF–LFb (Figure 4C), HF–HFb (Figure 4D), LF–LFb (Figure 4E), and LF–HFb (Figure 4F). Overall, high-fiber diets had more Firmicutes ASVs that were significantly increased in abundance following antibiotics, compared to low-fiber diets in which a large number Firmicutes-associated ASVs were significantly reduced in abundance following antibiotics. Taken together, these findings indicate a degree of resiliency among Firmicutes species when mice consume a high-fiber diet during antibiotic exposure.

### Fermentable fiber consumption increases acetyl-CoA production and induces LuxS/Autoinducer-2 within the microbiota in a manner independent of fat intake

**Figure 5.**
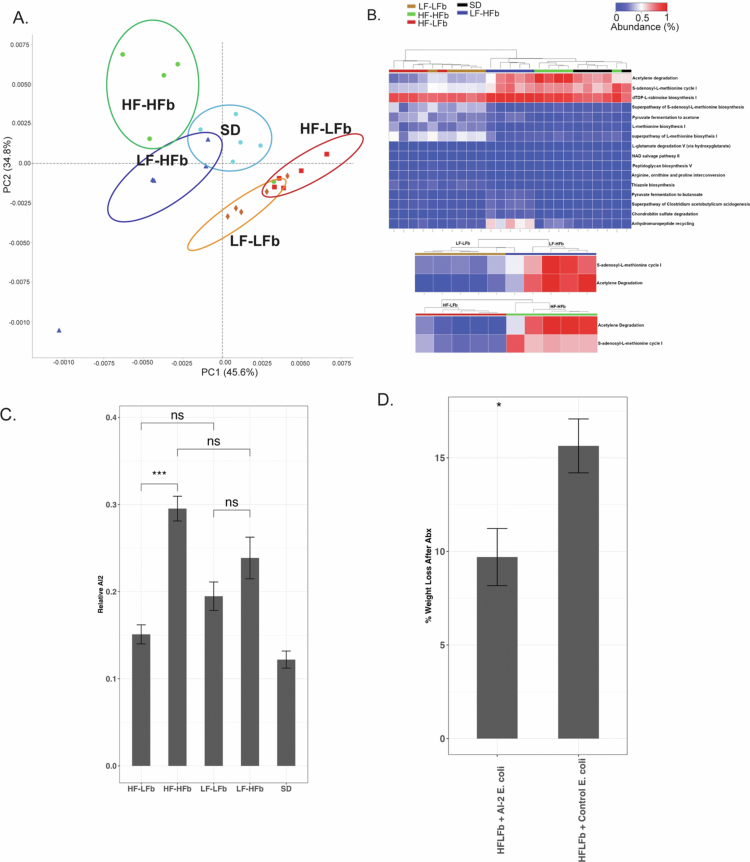
Functional prediction of 16S rRNA sequencing demonstrated significant differences in pathways determined by STAMP (A). On PICRUSt analysis, dietary fiber significantly increases acetyl-CoA production and the synthesis of the interspecies quorum-sensing molecule AI-2 (S-adenosyl-L-methionine cycle I). Heat map comparing the most significantly abundant pathways among all the diets (B, *p* < 0.05, FDR) along with comparison of the abundance of acetylene degradation and AI-2 synthesis between the low-fat (LF–LFb and LF–HFb) and high-fat (HF–LFb and HF–HFb) diets. The relative amount of AI-2 was measured utilizing a *Vibrio* AI-2 reporter strain and demonstrated a significant increase in HF–HFb compared to HF–LFb fed mice (C). *E. coli* that constitutively produces AI-2 was grown, and supernatant was collected and administered via enema one day prior to and during antibiotic administration and compared to the control *E. coli* supernatant that does not produce AI-2. The administration of AI-2 supernatant significantly reduced antibiotic-induced weight loss in HF–LFb-fed mice (D) *n* = 5 for all groups. *p* > 0.05 ns, *p < 0.05, **p < 0.01, ***p < 0.001, and ****p < 0.0001.

To further evaluate the differences in the functional profile of the intestinal microbiota between the different diets, PICRUSt and STAMP were utilized to perform functional predictions based on 16S rRNA amplicons of the stool and cecal microbiota. Similar to the findings evaluating microbiota composition, the functional profiles of the cecal microbiota were similar between the low-fiber and high-fiber diets (Figure 5A). In pairwise comparison between low-fiber diets (HF–LFb and LF–LFb), there was only a single significantly different pathway in the stool and 64 significant pathways in the cecum (Supplemental Figure 2A). When a pairwise comparison was performed between high-fiber diets (HF–HFb and LF–HFb), there were only 20 significantly different pathways in the stool, and no significantly different pathways were activated in the cecum (Supplemental Figure 2A,B). When high-fiber and low-fiber diets were compared, a greater number of significant functional pathways differed between the diets (1769 significant pathways, HF–HFb vs. HF–LFb and 703 significant pathways, LF–HFb vs. LF–LFb, *p* < 0.05 FDR). When the diets were compared to SD, the stool microbiota functional profile of the LF–HFb diet appeared to be the most similar to that of the SD diet, with only 26 significantly different pathways (957 vs. 1305 vs. 938 vs. 26; HF–HFb vs. HF–LFb vs. LF–LFb vs. LF–HFb, *p* < 0.05 FDR).

When the postantibiotic exposure functional profiles were compared, the microbiota functional profiles of the diets appeared to be more similar based on dietary fat content rather than fiber content. For example, after antibiotic treatment, HF–HFb demonstrated activation of 64 distinct pathways from those of HF–LFb, compared to 529 distinct pathways when HF–HFb was compared to LF–HFb. Similarly, there were only 90 distinct pathways between LF–HFb and LF–LFb. In summary, the dietary fiber content appeared to determine the functional profile of the stool and cecal microbiota prior to antibiotic treatment, and the dietary fat content appeared to determine the microbiota profile following antibiotic administration (Supplemental Figure 3).

The pathway differences prior to antibiotic exposure between the diets were further assessed, and the two pathways that were most significantly altered included acetylene degradation and the S-adenosyl-L-methionine cycle I (Figure 5B). Both of these pathways were significantly increased in high-fiber diets, independent of fat content (Figure 5B). Acetylene degradation is essential for the production of acetyl-CoA, which is important for the production of both butyrate and acetate, which may explain the increase in acetate and butyrate observed with the addition of fermentable fiber.^29^ Finally, the S-adenosyl-L-methionine pathway is associated with the production of quorum-sensing molecules utilizing LuxS/Autoinducer-2 (AI-2). AI-2 has been found to be produced by different species of bacteria and has been deemed a universal signaling molecule involved in interspecies communication.^30^ AI-2 has been found to play a role in biofilm formation, virulence, and antibiotic resistance.^30^ Particularly, increased production of AI-2 has been found to increase the resiliency of Firmicutes to antibiotic treatment.^28^ Fermentable fibers may be able to increase metabolites that provide important host benefits in the setting of antibiotic exposure while simultaneously increasing the ability of the microbiota communities to communicate and maintain resiliency in the face of antibiotics.

Given the association between AI-2 production and Firmicutes resiliency during antibiotic treatment, the production of AI-2 by the cecal microbiota of each diet group was measured utilizing an AI-2 reporter strain, *Vibrio campbelli*. There was a significant increase in relative AI-2 production in the HF–HFb microbiota compared to the HF–LFb microbiota (*p* < 0.01) and a nonsignificant increase in AI-2 production in the LF–HFb microbiota compared to the LF–LFb microbiota (Figure 5C). Furthermore, to test the sufficiency of AI-2 in preventing antibiotic-induced weight loss, HF–LFb diet-fed mice were administered an enema during the course of antibiotics that contained either supernatant collected from *E. coli* that constitutively expresses AI-2, or the supernatant collected from a control *E. coli* strain. When mice received enemas containing AI-2 supernatant, there was a significant reduction in antibiotic-induced weight loss (Figure 5D).

## Discussion

Independent of dietary fat, fermentable fiber has a major impact on both the intestinal microbiota composition and function and is associated with host resiliency and microbiota stability during antibiotic administration, as judged by decreased weight loss and diarrhea, as well as increased production of SCFAs and expression of AI-2 among high-fiber diets. These results suggest that dietary supplementation in the form of plant-based fiber may offer a method enhancing the resiliency of the microbiota with the goal of mitigating complications related to antibiotic exposure.

The intestinal microbiota plays an essential role in shaping the host immune system and preventing pathogen colonization. Exposure to antibiotics has a significant impact on the intestinal microbiota and can negatively impact the host immune system, leaving patients more vulnerable to pathogen colonization.^31-34^ Numerous studies have demonstrated that the loss of health promoting Bacteroidetes and SCFAs results in a significant increase in pathogen colonization, which in turn can result in further infectious complications.^35^^,^^36^ Here, we have shown that the addition of dietary fiber supplementation in both low- and high-fat diets increased Bacteroidetes abundance, reduced Proteobacteria abundance, and increased intestinal SCFAs, which together may contribute to host immune activation and prevent the colonization of pathogenic bacteria.

The combination of a Western diet with antibiotic exposure further disrupts the microbiota and leaves the host susceptible to both infectious and inflammatory diseases. A recent study demonstrated that the combination of a high-fat diet with antibiotics perturbs the intestinal microbiota in a manner that alters the mitochondrial activity of colonocytes, resulting in increased intestinal inflammation in a manner similar to that in patients with inflammatory bowel disease.^37^ Additionally, our recent study demonstrated that mice fed a Western diet, exposed to antibiotics, and subjected to surgical injury developed lethal gut-derived sepsis, whereas mice fed standard chow were protected against lethal sepsis. The addition of plant-based, fermentable fiber may offer an opportunity to improve the resiliency of the microbiota in the face of the selective pressures of antibiotics and dietary changes. This effect may be mediated, in part, by the production of SCFAs and quorum-sensing signaling via AI-2.^28^

Although the addition of microbiota-accessible fiber improved the intestinal microbiota and the host response to antibiotics, these high-fiber diets did not restore the microbiota to the same level as seen in the plant-based standard rodent chow diet. The high-fiber diets used in these experiments represent a refined diet with the addition of a single, plant-based fiber. Different plant-based fibers have been shown to result in species-specific variations within the intestinal microbiota; therefore, a food source such as standard rodent chow that contains a wide variety of unprocessed plant-based fibers will have a broader impact on the intestinal microbiota.^38^ Finally, microbiota diversity and resiliency have been found to be associated with increased dietary diversity.^39^ Utilizing a single fiber type may explain the reduction in alpha diversity in our study and the failure of fiber supplementation to restore microbiota resiliency similar to that of mice fed standard rodent chow. In humans, the physiologic and microbiota response to microbiota-accessible carbohydrates is dependent on the specific type of fiber that is utilized, and dietary diversity is important for maintaining a resilient microbiota.^39^^,^^40^ This is also likely exemplified by the inability of a single fiber to refine the diet to restore the gut microbiota to the level of standard rodent chow as standard rodent chow has a high diversity of unprocessed grains high in microbiota-accessible carbohydrates.^3^ Future studies may need to incorporate a variety of fiber sources to reach the microbiota levels and stability seen in standard rodent chow.

Our study is not without limitations, we utilized a single strain of mice from a single vendor, and the response of the intestinal microbiota to diet and antibiotics is likely influenced by the background genetics of the host. It is also important to understand that the microbiota response to dietary interventions is highly individualized,^39^ and not all patients may have the same response to dietary fiber as reported in this study. Additionally, for 16S sequencing, our sample sizes were relatively small and therefore were not powered to detect smaller differences in the gut microbiota. Finally, diets were consumed ad libitum, and pair feeding was not utilized in this study; therefore, the results could be a function of dietary quantity in addition to dietary quality.

Approximately 100,000 elective operations are performed in the US daily, of which virtually every patient receives a single dose of intravenous antibiotics, and selected patients may receive prolonged antibiotics even after an invasive procedure. Given that antibiotics are generally regarded as safe, their use among medical providers is promiscuous and frequent. In addition, most hospitalized patients are placed on chemically refined, sterile diets that are completely lacking plant-based fibers and have been shown to negatively impact the intestinal microbiota and metabolome.^10^ Therefore, advancing a greater understanding of the impact of diet and antibiotics on the microbiome and their downstream effects on the host may be warranted. The results of this study suggest that the use of plant-based fibers to fortify diets may strengthen the resiliency of the intestinal microbiota in the face of antibiotic use and mitigate the subsequent infection risks associated with antibiotic exposure.

## Supplementary Material

Supplemental_Figure3.jpg Supplemental_Figure3.jpg

Supplemental_Figure1.jpg Supplemental_Figure1.jpg

Supplemental_Figure_2.jpg Supplemental_Figure_2.jpg

Supplemental Material Supplemental_Figure_caption.docx

## Data Availability

16S sequencing is available through NCBI SRA (reference number: PRJNA83864, metadata supplemental file 1). Additional data are available upon request.
