# Supplementary figures and images for: Microbiota-accessible carbohydrates enhance gut microbiota stability and antibiotic resilience through production of quorum sensing molecule AI-2
Source: Gut Microbes Rep. 2026 Mar 23;3(1):2646055. doi: 10.1080/29933935.2026.2646055 (PMC13034624; doi:10.1080/29933935.2026.2646055)

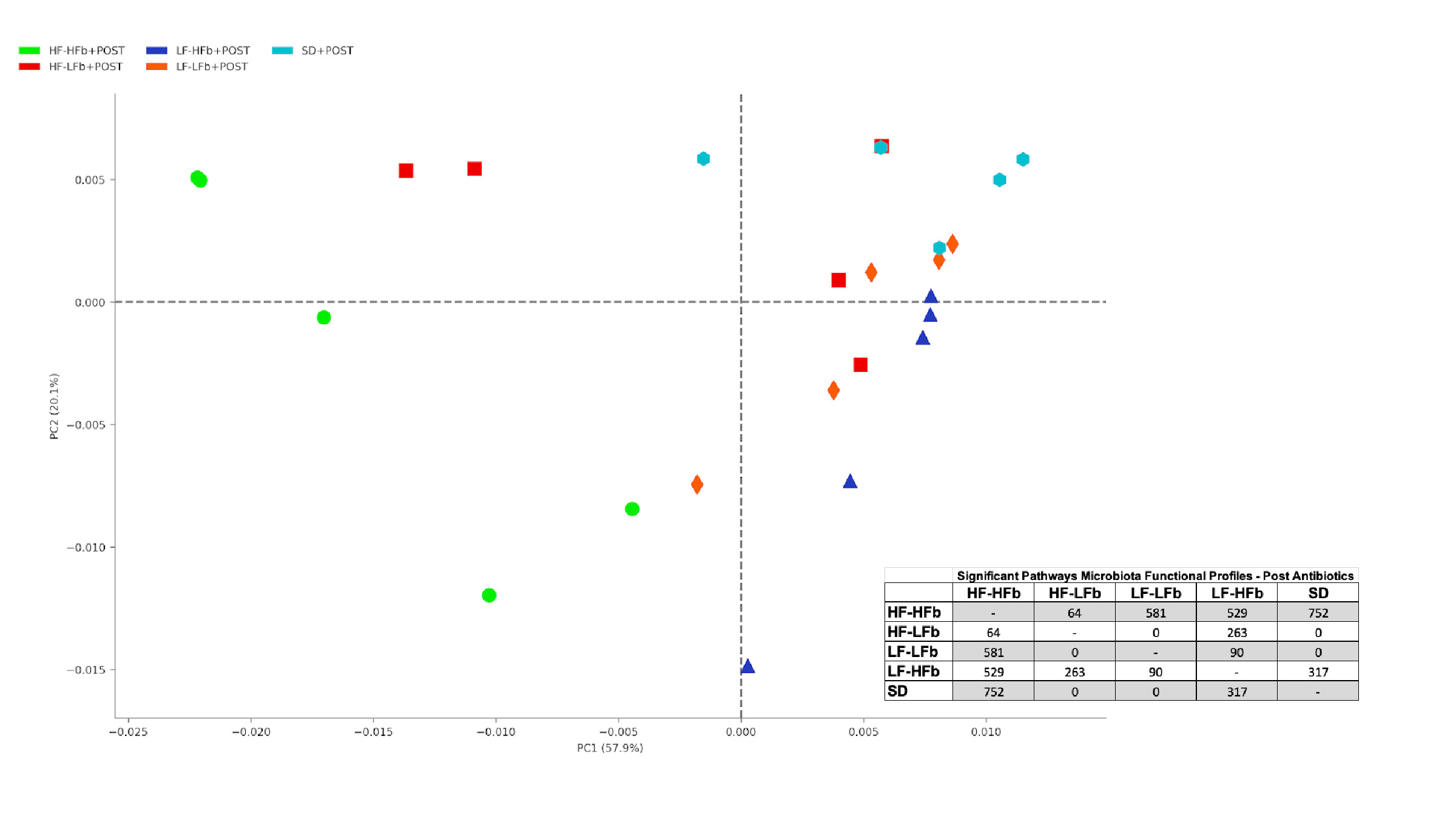

Supplement: Supplemental_Figure3.jpg [file KGMR_A_2646055_SM7931.jpg]

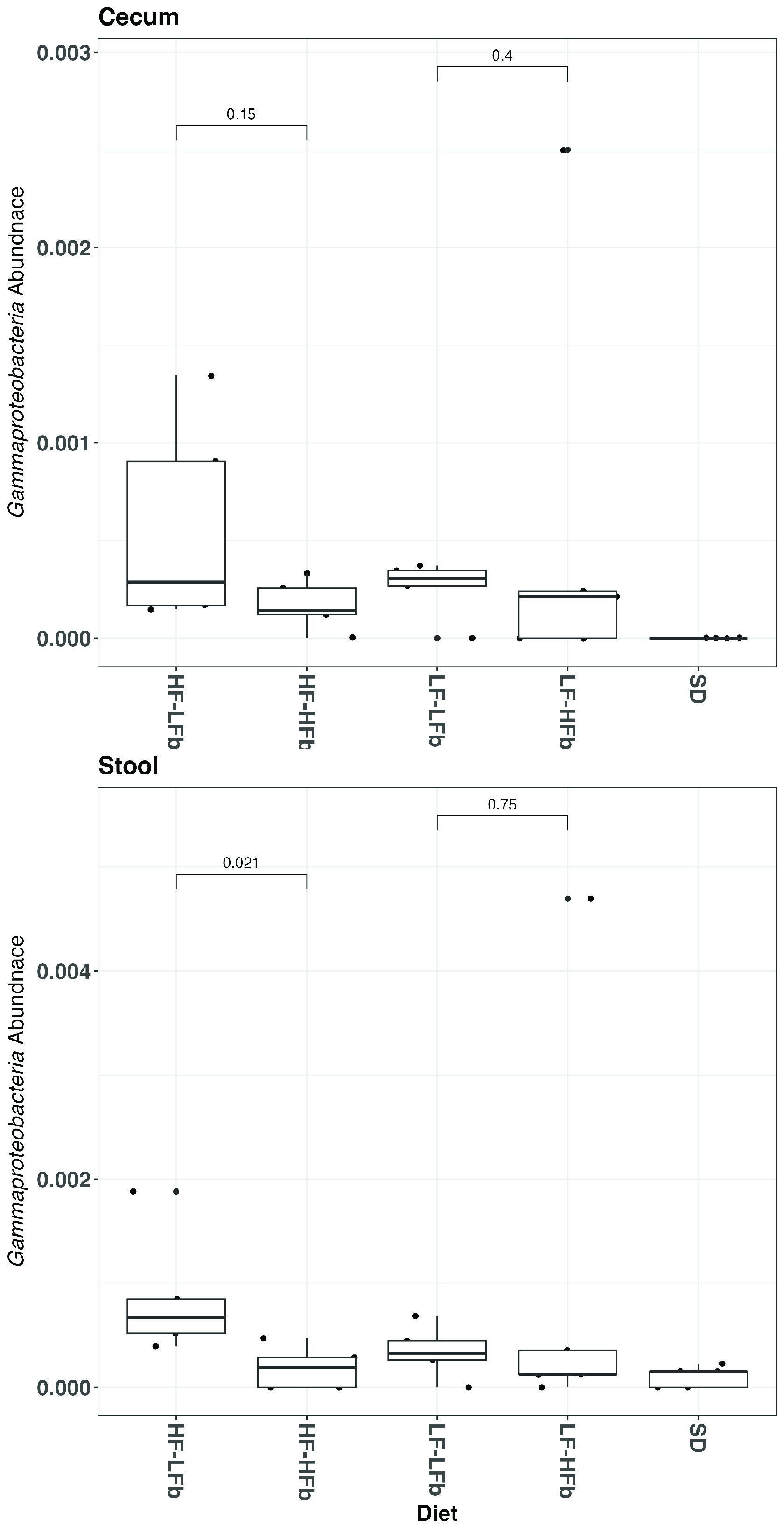

Supplement: Supplemental_Figure1.jpg [file KGMR_A_2646055_SM7929.jpg]

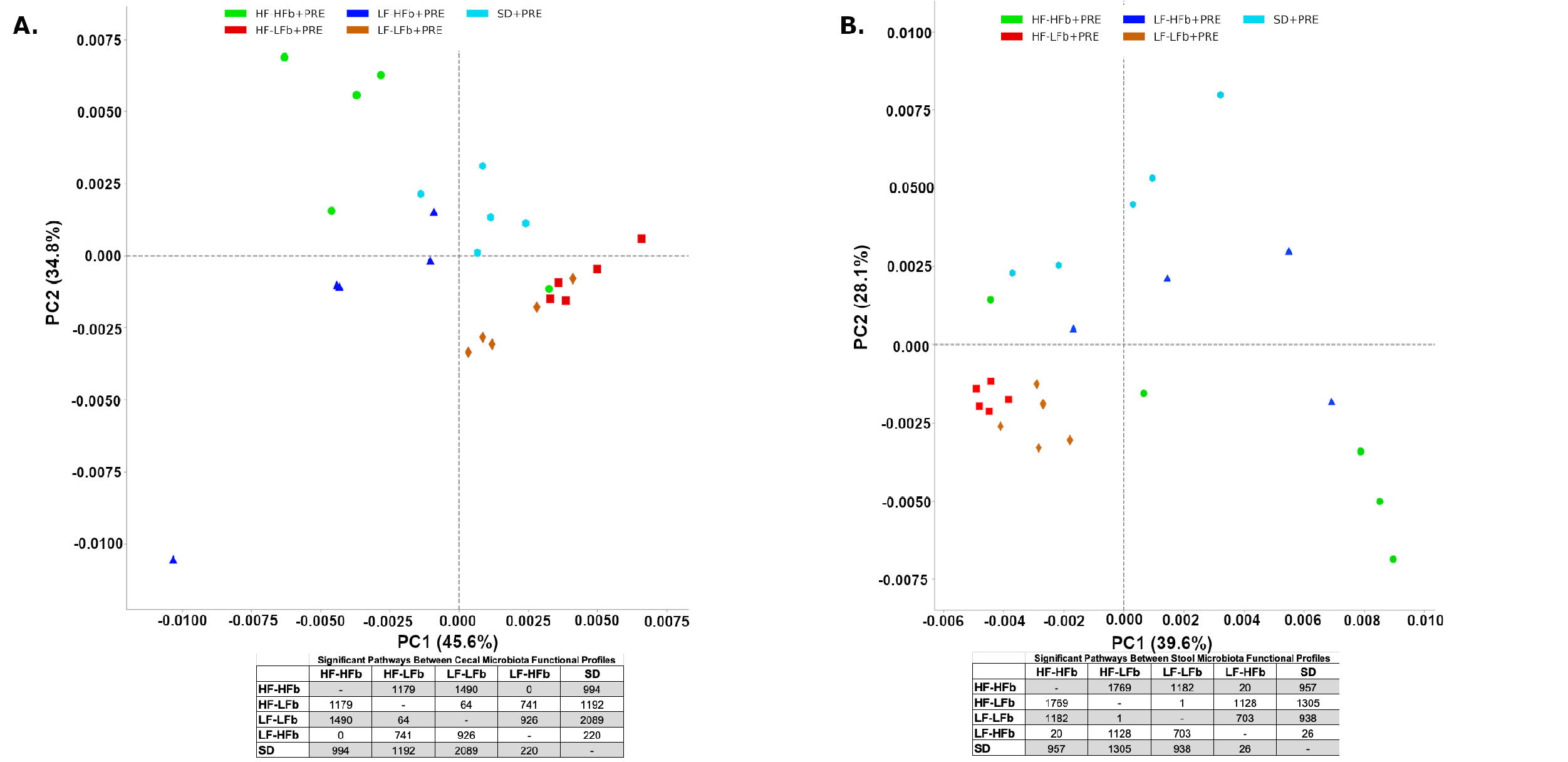

Supplement: Supplemental_Figure_2.jpg [file KGMR_A_2646055_SM7930.jpg]
