# Supplementary material for: Microbiota-accessible carbohydrates enhance gut microbiota stability and antibiotic resilience through production of quorum sensing molecule AI-2
Source: Gut Microbes Rep. 2026 Mar 23;3(1):2646055. doi: 10.1080/29933935.2026.2646055 (PMC13034624; doi:10.1080/29933935.2026.2646055)
Supplement: Supplemental Material — Supplemental_Figure_caption.docx [file KGMR_A_2646055_SM1265.docx]

**Supplemental Figure 1:** Abundance of *Gammaproteobacteria* in the cecum and stool.

**Supplemental Figure 2:** Ordination plots demonstrating differences in functional pathway analysis utilizing STAMP of the cecal (A) and stool (B) microbiota of their respective diets prior to antibiotic exposure. The tables beneath the plots represent significantly different pathways between the diets utilizing pairwise comparisons and Benjamini-Hochberg correction (p < 0.05).

**Supplemental Figure 3:** Ordination plots of the predicted pathways based on STAMP analysis of the cecal microbiota post antibiotic exposure. The table represents significantly altered pathways between the diets on pairwise comparison utilizing Benjamini-Hochberg correction (p < 0.05)
